# Role of tillage measures in mitigating waterlogging damage in rapeseed

**DOI:** 10.1186/s12870-023-04250-7

**Published:** 2023-05-01

**Authors:** Xiaoqin Tian, Zhuo Li, Yonghong Liu, Wei Li

**Affiliations:** 1 Crop Research Institute, Sichuan Academy of Agriculture Sciences, Chengdu, 610066 China; 2 Provincial Key Laboratory of Water-Saving Agriculture in Hill Areas of Southern China, Chengdu, 611100 China; 3 Environment-friendly Crop Germplasm Innovation and Genetic Improvement Key Laboratory of Sichuan Province, Chengdu, 610066 China; 4 Sichuan Academy of Agriculture Sciences, Chengdu, 610066 China; 5 Sichuan Huabiao Testing Technology Co., Ltd., Chengdu, 611731 China

**Keywords:** Rapeseed, Waterlogging stress, Ridge, Yield, Film side, Antioxidant enzyme

## Abstract

**Background:**

Tillage measures have been effectively adopted for mitigating waterlogging damage in field crops, yet little is known about the role of tillage measures in crop responses to waterlogging. A field experiment was performed to investigate the effect of conventional planting (CK), small ridge planting (SR), big ridge planting (BR) and film side planting (FS) on soil available nutrients and enzymatic activity, chlorophyll contents, leaf nutrients, soluble protein, soluble sugar, nitrate reductase, antioxidant enzyme activity, lipid peroxidation, agronomic traits and yield of rapeseed under waterlogging stress conditions.

**Results:**

Tillage measures remarkably improved rapeseed growth and yield parameters under waterlogging stress conditions. Under waterlogging conditions, rapeseed yield was significantly increased by 33.09 and 22.70% in the SR and BR groups, respectively, compared with CK. Correlation analysis showed that NO_3_^−^-N, NH_4_^+^-N, and urease in soils and malonaldehyde (MDA), superoxide dismutase (SOD), and nitrate reductase in roots were the key factors affecting rapeseed yield. The SR and BR groups had significantly increased NO_3_^−^-N by 180.30 and 139.77%, NH_4_^+^-N by 115.78 and 66.59%, urease by 41.27 and 26.45%, SOD by 6.64 and 4.66%, nitrate reductase by 71.67 and 26.67%, and significantly decreased MDA content by 14.81 and 13.35% under waterlogging stress, respectively, compared with CK. In addition, chlorophyll and N content in leaves, soluble sugar and POD in roots, and most agronomic traits were also significantly enhanced in response to SR and BR under waterlogging conditions.

**Conclusion:**

Overall, SR and BR mitigated the waterlogging damage in rapeseed mainly by reducing the loss of soil available nitrogen, decreasing the MDA content in roots, and promoting urease in soils and SOD and nitrate reductase in roots. Finally, thorough assessment of rapeseed parameters indicated that SR treatment was most effective followed by BR treatment, to alleviate the adverse effects of waterlogging stress.

## Background

Rapeseed (*Brassica napus* L.) is an important oil crop, as well as an important protein and energy crop [1]. In China, rapeseed is the most important oil crop, and Sichuan is one of the main rapeseed producing areas. The sown area and total output of rapeseed in Sichuan were estimated to be 1.29 million ha and 3.17 million tons, respectively, in 2020, ranking first in China [2]. “Looks at Sichuan for rapeseed” has become a consensus, and doing a good job in the production of rapeseed in Sichuan is strategically important to ensure the safety of edible vegetable oil in Sichuan and China. Rice-rapeseed rotation is one of the main crop rotations in Sichuan. Waterlogging at the seedling stage of rapeseed often occurs due to continuous rain every autumn, high groundwater levels and large soil viscosity [3]. The seedling stage of rapeseed is an important period for the population, which is dominated by vegetative growth. There is a close relationship between plant growth and rapeseed resistance and late yield [4]. Previous studies have shown that of rapeseed is an oil crop sensitive to waterlogging stress, especially during critical growth periods (e.g. seedling stage) [5]. Waterlogging at the seedling stage of rapeseed causes various changes in crop plants through different morphological, physiological, and biochemical responses [6]. For example, root tissue necrosis, significant reduction of root biomass, and significant changes in root morphology have been observed [3]. Moreover, chlorophyll content, photosynthetic rate, superoxide dismutase (SOD), peroxidase (POD), catalase (CAT) and root activity significantly decreased [7]. Therefore, it is important to explore reasonable and effective cultivation measures to alleviate waterlogging stress in rapeseed at the seedling stage to realize higher rapeseed yields.

Currently, the measures to mitigate waterlogging damage or improve waterlogging tolerance in crop production mainly include breeding and selecting crop varieties with strong moisture tolerance [8], ditching and drainage to reduce groundwater levels [9], and taking remedial measures such as water-fertilizer regulation [7] or chemical regulation [3] after waterlogging stress. Among them, the first two measures are time-consuming and laborious, and the latter are costly and may affect crop quality and the environment. In contrast, tillage is an important technology to restore the growth of waterlogged crops, which can alleviate the damage of waterlogging stress to plant growth to a certain extent and is easy to implement. A previous study showed that the light transmittance of the ear layer in the ridge tillage treatment for DH605 and ZD958 was decreased by 12 and 19% at the tasselling stage, respectively, compared with that of the waterlogging treatment [10]. Visibly, ridge tillage effectively alleviated leaf senescence and the decrease in leaf area index (LAI) and chlorophyll content induced by waterlogging and improved canopy structure, photosynthetic effective radiation, and photosynthesis of waterlogged summer maize, thus increasing grain yield by 39 and 50% for DH605 and ZD958, respectively, compared with waterlogging treatments [10]. Similarly, Du et al. [11] reported that a raised bed planting pattern significantly increased soil water drainage and reduced the soil water content. The reduced waterlogging stress promoted wheat seedling establishment and root growth, accelerated stem and tiller development, and delayed late-season root and leaf senescence, resulting in 11.3 and 14.1% higher grain yields in Fengtai and Guohe, respectively. In addition, Suo et al. [12] recorded that ploughing to a depth of 22 cm was superior to ploughing to a depth of 18 cm for waterlogged paddy rice yield.

The current study on the waterlogging of rapeseed mainly focused on the evaluation of seed moisture tolerance and the response of seedlings to waterlogging in terms of morphological and physiological indicators. Research on the control measures of waterlogging of rapeseed in the seedling stage is still rare, and no investigation has been carried out to reveal the mechanism of mitigating waterlogging damage in rapeseed by control measures from soil indicators and plant indicators. The objectives of the study were to (1) determine the effect of different tillage measures on soil available nutrients and enzymatic activity and rapeseed chlorophyll contents, leaf nutrients, soluble protein, soluble sugar, nitrate reductase, antioxidant enzyme activity, lipid peroxidation, agronomic traits and yield under waterlogging stress conditions; (2) compare the different tillage measures to alleviate the harmful effects of waterlogging stress; and (3) explain the mechanism by which tillage measures alleviate waterlogging stress.

## Results

### Chlorophyll and nutrient content in leaves

**Fig. 1:**
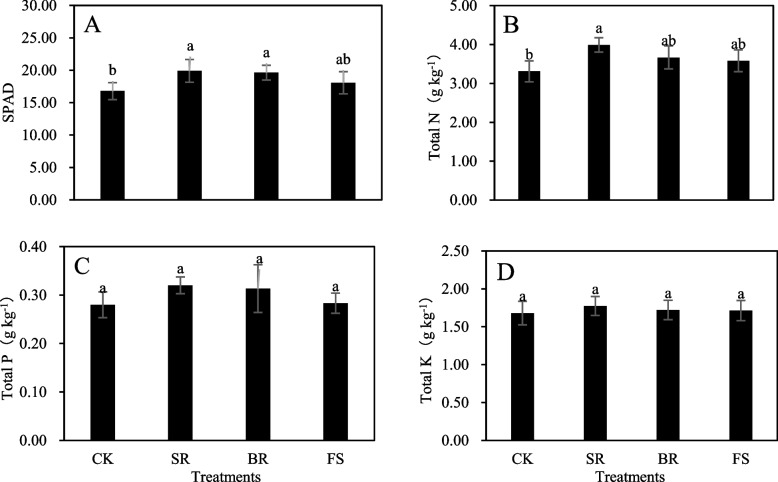
Comparative of chlorophyll, nutrient elements in rapeseed leaves under different tillage measures after release of waterlogging tolerance. Different small letters indicate significant differences among treatments, at *P* < 0.05 level. Each value is the mean ± SD of three replicate measurements. CK, conventional planting; SR: small ridge planting; BR: big ridge planting; FS: film side planting

Tillage measures statistically (*P* < 0.05) affected the chlorophyll (except for FS) and N contents (except for BR and FS) in leaves (Fig. 1A, B) but not the P and K contents in leaves (Fig. 1C, D). The results showed that the chlorophyll and nutrient contents in leaves were improved by the SR, BR, and FS treatments. SR treatment significantly increased the chlorophyll and N contents in leaves by 18.59 and 20.54%, respectively, and BR treatment significantly enhanced chlorophyll by 16.96% compared with the values of the CK treatment (Fig. 1A, B). Overall, maximum chlorophyll and nutrient contents in leaves were recorded from the plants treated with SR followed by BR and FS treatments compared with the CK treatment.

### Available nitrogen content and enzyme activity in soil

**Fig. 2:**
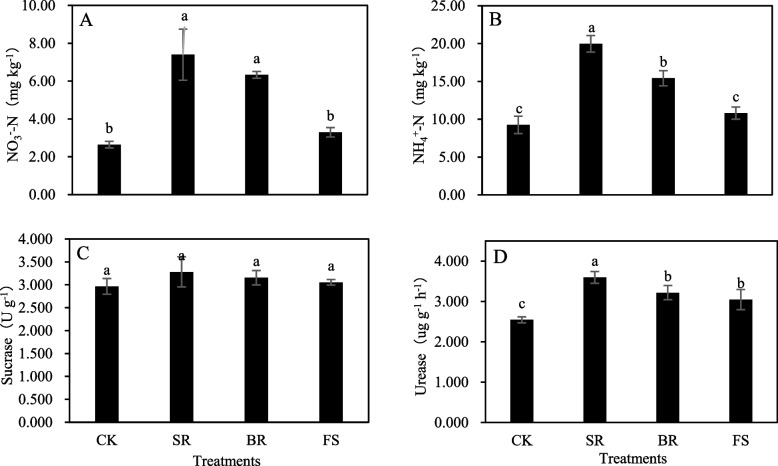
Comparative of available N, enzyme activity in soil under different tillage measures after release of waterlogging tolerance. Different small letters indicate significant differences among treatments, at *P* < 0.05 level. Each value is the mean ± SD of three replicate measurements. CK, conventional planting; SR: small ridge planting; BR: big ridge planting; FS: film side planting

NO_3_^−^-N, NH_4_^+^-N and urease in soil were significantly (*P* < 0.05) affected by the tillage measures (Fig. 2A, B, D), while sucrase in soil was less affected by the tillage measures (Fig. 2C). Nevertheless, the available nitrogen content and enzyme activity in soil were enhanced by the SR, BR, and FS treatments. NO_3_^−^-N and NH_4_^+^-N were significantly impacted by the SR and BR treatments, and urease was significantly influenced by the SR, BR, and FS treatments (Fig. 2A, B, D). The respective treatments improved NO_3_^−^-N by 180.30 and 139.77%, NH_4_^+^-N by 115.78 and 66.59%, and urease by 41.27, 26.45 and 19.73%, respectively, compared with the values of the CK treatment. Generally, the highest available nitrogen content and enzyme activity in soil were recorded from the plants treated with SR, followed by the BR and FS treatments compared with the CK treatment.

### Soluble protein, sugar, and nitrate reductase in roots

**Fig. 3:**
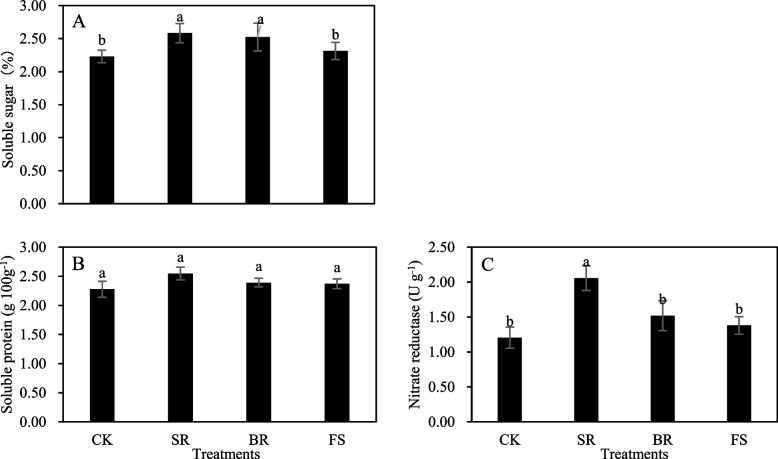
Comparative of metabolism in rapeseed roots under different tillage measures after release of waterlogging tolerance. Different small letters indicate significant differences among treatments, at *P* < 0.05 level. Each value is the mean ± SD of three replicate measurements. CK, conventional planting; SR: small ridge planting; BR: big ridge planting; FS: film side planting

Soluble sugar and nitrate reductase were statistically (*P* < 0.05) influenced by the tillage measures (Fig. 3A, C), while soluble protein was less affected by the tillage measures (Fig. 3B). The results showed that soluble protein, sugar, and nitrate reductase in roots were increased by the SR, BR, and FS treatments. Soluble sugar was significantly affected by the SR and BR treatments, and nitrate reductase was only significantly impacted by the SR treatment (Fig. 3A, C). The respective treatments promoted the soluble sugar by 15.70 and 13.00% and nitrate reductase by 71.67% compared with the values of the CK treatment. In general, the highest soluble protein, sugar, and nitrate reductase in roots were registered from the plants treated with SR followed by BR and FS treatments compared with the CK treatment.

### Antioxidant enzyme activities and lipid peroxidation in roots

**Fig. 4:**
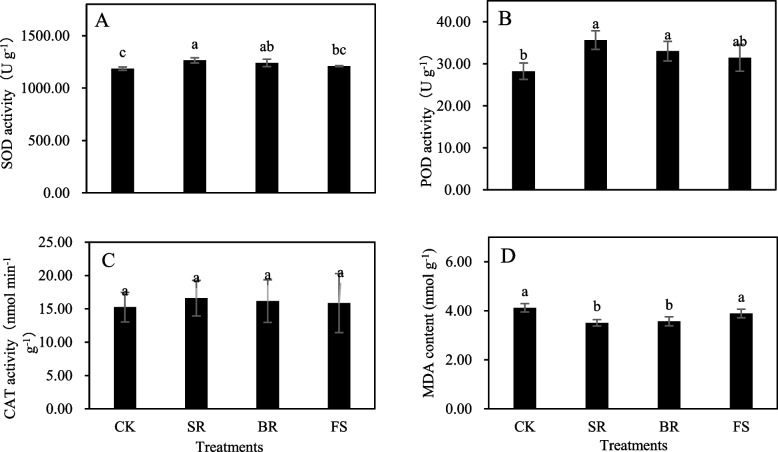
Comparative of physiology in rapeseed roots under different tillage measures after release of waterlogging tolerance. Different small letters indicate significant differences among treatments, at *P* < 0.05 level. Each value is the mean ± SD of three replicate measurements. CK, conventional planting; SR: small ridge planting; BR: big ridge planting; FS: film side planting

Significant (*P* < 0.05) effects of tillage measures were found for SOD and POD (except for FS) and malonaldehyde (MDA) content in roots (Fig. 4A, B, D), and no effect of tillage measures was found for the CAT activity in roots (Fig. 4C). The results indicated that SOD, POD and CAT activities were increased and MDA content was decreased by SR, BR and FS treatments. SOD and POD activities and MDA contents were statistically impacted by SR and BR treatments (Fig. 4A, B, D). The respective treatments improved the activity of SOD by 6.64 and 4.66% and POD by 26.24 and 16.93% but reduced the MDA content by 14.81 and 13.35%, respectively, compared with the values of the CK treatment. Overall, the highest antioxidant enzyme activities and the lowest MDA contents in roots were recorded from the plants treated with SR, followed by the BR and FS treatments.

### Yield and agronomic traits

Tillage measures significantly (*P* < 0.05) influenced yield and agronomic traits in terms of H (except for FS and BR), EBH, MSH (except for FS), OEB (except for FS), OEP and GP (except for FS and BR). The results indicated that yield and agronomic traits increased with SR, BR and FS treatments (Table 1). As expected, SR treatment significantly enhanced all yield parameters except for MSP and TGW compared with CK. The respective treatments increased H by 8.60%, EBH by 26.96%, MSH by 16.01%, OEB by 31.58%, OEP by 76.87%, GP by 19.30% and yield by 33.09% compared with the values of the CK treatment. In addition, the BR treatment also significantly enhanced EBH by 21.47%, MSH by 15.05%, OEB by 28.07%, OEP by 57.46% and yield by 22.70% compared with the values of the CK treatment. Overall, the highest yield parameters were recorded from the plants treated with SR, followed by the BR and FS treatments, compared with the CK treatment.

**Table 1:** Comparative of agronomic traits and yield in rapeseed under different tillage measures after waterlogging tolerance

Treatments	H (cm)	EBH (cm)	MSH (cm)	OEB (pcs)	OEP (pcs)	MSP (pcs)	GP (pcs)	TGW (g)	Yield (kg hm^−2^)
CK	157.27 ± 5.63b	94.73 ± 7.10c	62.47 ± 3.90b	5.7 ± 0.3b	134 ± 11d	73 ± 6a	17.1 ± 1.4b	4.55 ± 0.08a	1354.08 ± 126.58b
SR	170.80 ± 4.73a	120.27 ± 4.64a	72.47 ± 5.75a	7.5 ± 0.6a	237 ± 18a	80 ± 2a	20.4 ± 0.3a	4.86 ± 0.21a	1802.19 ± 82.18a
BR	168.87 ± 9.20ab	115.07 ± 11.87ab	71.87 ± 5.18a	7.3 ± 1.1a	211 ± 14b	78 ± 6a	19.3 ± 1.9ab	4.71 ± 0.22a	1661.49 ± 80.05a
FS	162.07 ± 4.87ab	103.87 ± 2.50bc	67.07 ± 4.41ab	6.4 ± 0.2ab	160 ± 10c	75 ± 5a	18.6 ± 1.5ab	4.64 ± 0.17a	1399.94 ± 118.54b

Different small letters indicate significant differences among treatments, at *P* < 0.05 level. Each value is the mean ± SD of three replicate measurements

*H* plant height, *EBH* effective branching height, *MSH* main sequence height, *OEB* one effective branches, *OEP* one effective pods, *MSP* main sequence pods, *GP* grains per pod, *TGW* thousand-grain weight, *CK* conventional planting, *SR* small ridge planting, *BR* big ridge planting, *FS* film side planting

### Relationships between yield and variables

**Fig. 5:**
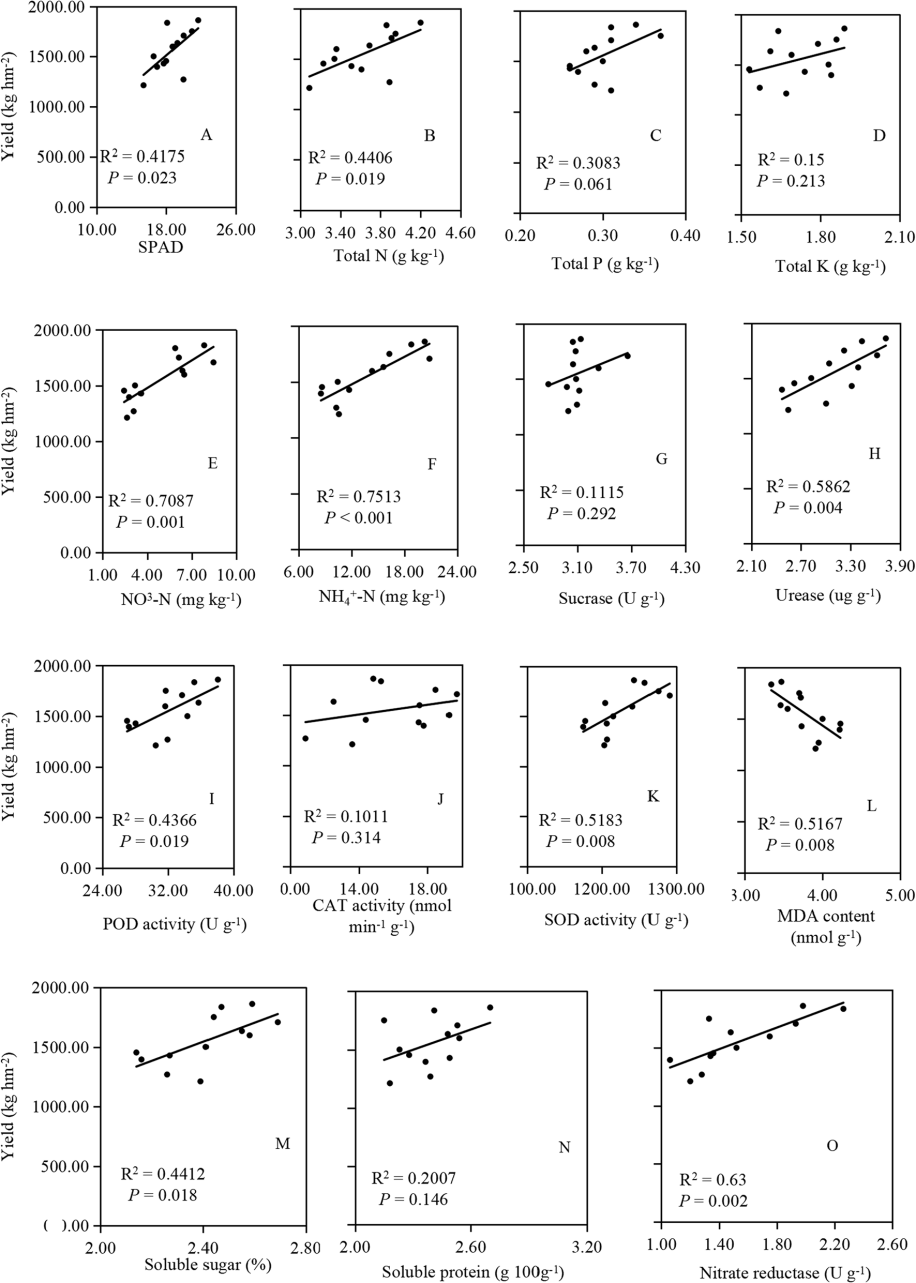
Relationships between yield and the physiological metabolic traits across different cultivation measures

Across different tillage measures, without close relationships with yield and total P, total K, sucrase, CAT and soluble protein (Fig. 5C, D, G, J, N), yield was significantly negatively correlated with MDA content, but was positively correlated with SPAD, total N, NO_3_^−^-N, NH_4_^+^-N, urease, POD, SOD, soluble sugar and nitrate reductase (Fig. 5A, B, E, F, H, I, K, L, M, O). Among the traits assessed, NO_3_^−^-N, NH_4_^+^-N, urease in soils, and MDA, SOD, nitrate reductase in roots were the strongest determinants of yield (R^2^ = 0.5167 ~ 0.7513). Relatively, the correlations of SPAD, total N in leaves, POD, soluble sugar in roots with yield were much lower (R^2^ = 0.4175 ~ 0.4412).

## Discussion

Waterlogging is a critical agricultural hazard, resulting in serious crop yield reduction [13]. However, ridge tillage led to lower declines in one effective branches and one effective pods that induced by waterlogging, resulting in an increased grain yield compared to flat tillage, with an average increase in yield of 27.90% (Table 1). This result was similar to that of previous work [14–16]. Total N is a beneficial variable to assess the nutritional status of plant leaves, which is closely related to nitrogen utilization efficiency and crop yield [17]. Our study also showed that ridge tillage effectively alleviated the decline in N content in leaves induced by waterlogging (Fig. 1B), which was conducive to supplying enough nutrients to reproductive growth in later stages for waterlogged rapeseed. This alleviation contributed to the increase of one effective branches and one effective pods, ultimately resulting in an increased grain yield of waterlogged rapeseed. This result was consistent with that of a previous study [15, 18].

In this study, the ridge promoted N content in leaves under waterlogging conditions could be ascribed to the reduction in soil available nitrogen loss (Fig. 2A, B) and the improvement in urease activities in soils and nitrate reductase activities in roots, thus transporting more effective N into leaves. A decrease in effective N content in soil as one of the most substantial restricting factors for plant nutrition under abiotic stress have been documented by numerous other studies [19–22]. Our results indicated that NO_3_^−^-N, NH_4_^+^-N and urease in soil were significantly enhanced by ridges under waterlogging conditions, compared with flat conditions, because ridges could decrease N denitrification, leaching and runoff and reduce soil nitrogen mineralization rates [21].

Chlorophyll is the main plant photosynthetic pigment and plays an important role in rapeseed yield. The decreases in photosynthetic pigments, as one of the most substantial restricting factors for plant photosynthetic activity under abiotic stress have been documented by numerous other studies [23–26]. The SPAD value is a very powerful parameter to measure the relative chlorophyll content or green degree. Our results indicated that SPAD in leaves was significantly increased by ridges under waterlogging stress compared with flat leaves (Fig. 1A). This result showed that ridge tillage was conducive to alleviating the decline in chlorophyll content induced by waterlogging and thus delayed leaf senescence, resulting in the improvement of photosynthetic performance and ultimately increasing the grain yield of waterlogged rapeseed.

The reactive oxygen species (ROS) scavenging system plays an important role in protecting cells from photooxidative damage, and diverse enzymatic antioxidants can maintain the equilibrium between the production and scavenging of ROS, thus mitigating membrane peroxidation and decreasing the degree of oxidative damage induced by abiotic stresses [27–29]. SOD is considered the first line of defence against ROS accumulation, which stimulates the transformation of O_2_^−^ to O_2_ and H_2_O_2_ [30]. MDA content, an important indicator, reflects the degree of membrane lipid peroxidation [31]. In our study, ridges significantly increased the POD and SOD activities and decreased the MDA content under waterlogging conditions, compared with flat slopes (Fig. 4A, B, D). This finding indicated that ridges could effectively reduce the damage of waterlogging to the root antioxidant system, remove reactive oxygen species within a certain range in a timely manner, help to improve soil root activity and delay root senescence, and ultimately alleviate waterlogging damage to the growth and grain yield of rapeseed. In addition to the enzymatic defence system, some regulatory substances vigorously participate in the amelioration of waterlogging stress. Soluble sugar and soluble protein are very important for the osmoregulation process in plants under waterlogging stress. In this study, soluble sugar was also significantly increased by ridges (Fig. 3A). This phenomenon can be considered a portion of the mechanism to mitigate waterlogging damage in plants by adjusting the osmotic condition [32].

Growth and yield increases by ridges under waterlogging conditions are outside indicators of metabolic alterations in plant cells. In the past, many studies have indicated the effects of ridges on the growth performance and yields of various grain crops under waterlogging conditions [10–12, 14–16]. However, ridges alleviate the damage caused by waterlogging of crops as a result of the joint action of soil conditions and crop responses [15]. In addition, the damage range under waterlogging stress differed with the strength of stresses and the crop growth stages [33]. As noted in this study, all relevant results supported that ridges could be regarded as cultivation measures against nutrient loss and physiological and metabolic activity damage caused by waterlogging stress [19, 20], which could increase the effective N content and enzymatic activity in soil, improve the capacity of the antioxidant defence system, increase osmolyte accumulation, and decrease the MDA contents in waterlogging stress plant roots, thus ridges can be considered an important strategy to improve plant growth and yield attributes under waterlogging stress.

## Conclusions

Ridge tillage (planting rapeseed at a height of 20 cm above the ground) alleviated the negative effects of waterlogging on rapeseed by a joint action of soil conditions and crop responses, in which available nitrogen and urease activity in soil and nitrate reductase activity, SOD activity and MDA content in roots played major roles. Loss of available nitrogen was significantly reduced, urease activity was promoted in soil, nitrate reductase activity and SOD activity were significantly increased, and MDA content was decreased in roots by ridge tillage. In addition, ridge tillage also improved chlorophyll and N content in leaves, soluble sugar and POD in roots, and most agronomic traits. As a result, the grain yield of waterlogged rapeseed was significantly increased. Among the tillage measures, SR was most effective, followed by BR, in promoting the growth and yield attributes of rapeseed under waterlogging conditions. We should consider the employment of ridge tillage for sustainable agriculture in the future.

## Methods

### Plant material and experimental site

Seeds of rapeseed (Dexinyou-12, a locally adopted high-yielding rapeseed variety) were purchased from Chengdu Xingda Seed Industry Co., Ltd., Chengdu City, Sichuan Province, China, The field experiment was conducted from October 2020 to May 2021 at Jianyang Experimental Station (30°40′ N, 104°55′ E; elevation 460 m), Sichuan Academy of Agriculture Sciences, China. This experimental station is located in the eastern part of Sichuan basin in China, which has a typical subtropical monsoon climate. The average annual air temperature was 17 °C, and the average annual precipitation was 874 mm. According to the classification of the World Reference Base for Soil Resources the soil at the location of the field experiment can be classified as calcaric. The soil properties of the top 20 cm were as follows: pH: (1:2.5 soil: water), 6.6; organic matter, 16.3 g kg^− 1^; total N, 0.1%; total P, 0.8 g kg^− 1^; total K, 33.2 g kg^− 1^; available N, 247.0 mg kg^− 1^; available P, 25.0 mg kg^− 1^; available K, 98.7 mg kg^− 1^; which were determined by the conventional chemical analysis methods.

### Experimental design and field management

**Fig. 6:**
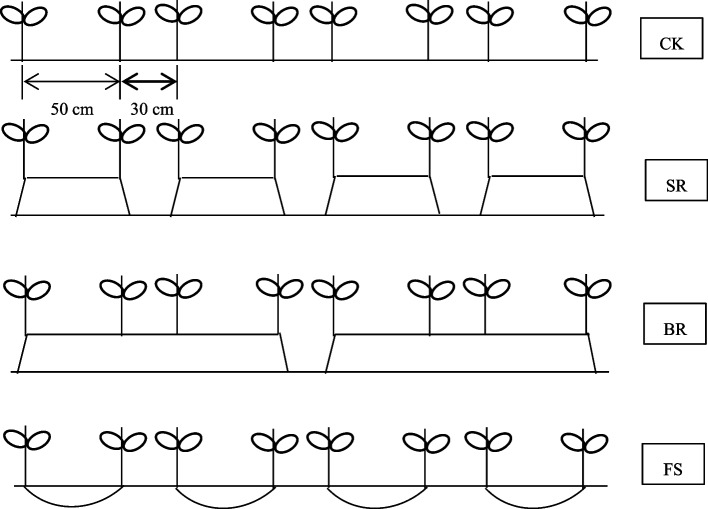
Planting diagram of rapeseed under different tillage measures

The field experiment was arranged in a randomized complete block design with four different tillage measures replicated three times. The four measures were (1) conventional planting (CK); (2) small ridge planting (SR); (3) big ridge planting (BR); (4) film side planting. Specific planting plan and planting diagram were given in Table 2 and Fig. 6, respectively. Individual plot was 20 m^2^ (4 m × 5 m) and each plot was separated by a 50-cm-wide ridge as a barrier. Besides, plastic film was used to isolate water in the 0–100 cm soil layer of each plot to avoid lateral water infiltration. Before planting, each treatment received 180 kg ha^− 1^ of Meifeng compound fertilizer (N-P_2_O_5_-K_2_O:18–16-18). Rapeseed (cv. “Dexinyou 12”) was planted by direct seeding on 8 October 2020 and harvested on 1 May 2021. At the 5–6 leaf stage of rapeseed, artificial rainfall was used until the water surface height was 1–2 cm above the surface soil layer of CK for 6 days, and the irrigation amount of other treatments was the same as CK.

**Table 2:** Experiment measures of rapeseed under different tillage measures

Treatments	Measures
CK	Conventional planting, wide line: narrow line = 50 cm: 30 cm
SR	Artificial ridge, ridge wide: ridge height = 60 cm: 20 cm, and 2 rows rapeseed were planted in ridges, wide line: narrow line = 50 cm: 30 cm.
BR	Artificial ridge, ridge wide: ridge height = 140 cm: 20 cm, and 4 rows rapeseed were planted in ridges, wide line: narrow line = 50 cm: 30 cm.
FS	Wide line: narrow line = 50 cm: 30 cm, and polyethylene film plastic mulch (colorless, transparent, 0.008 mm thick) covered the wide line, which was depressed.

### Soil sampling and analysis

Soil samples (0 ~ 20 cm) between plants by five sampling points were collected from each plot when waterlogging stress was relieved (after 6 days of CK waterlogging). During sampling and transportation, all the samples were kept in an insulated box with ice. By dividing each soil sample into two subsamples, one subsample was ground, passed through a 2-mm sieve and was air-dried for the analyses of sucrase and urease, and another one was ground, passed through a 2-mm sieve and was stored in a refrigerator at 4 °C for the analyses of NO_3_^−^ and NH_4_^+^. Sucrase was measured by 3,5-dinitrosalicylic acid colorimetric determination method [34]; Urease was measured by phenol-sodium hypochlorite indophenol colorimetry method [34]; The NO_3_^−^-N and NH_4_^+^-N were extracted with 2.0 M KCl and measured by a continuous flow analyzer (Flowsys, Systea Inc., Italy) [35].

### Plant sampling and analysis

When waterlogging stress was relieved, the chlorophyll content in the leaves was directly measured with a SPAD-502 Plus chlorophyll meter (Konica Minolta Holdings, Inc.), a single leaf was measured three times and 5 ~ 7 plants was measured in each plot [36]. After that, plant samples divided into leaves and roots were collected and cleaned. Leaves were dried at 70 °C for 48 h to constant weight and then finely ground into powder to pass through a 0.2 mm sieve, to determine the total N and *P* values using the flow analyzer (Flowsys, Systea Inc., Italy) and the total K values using the flame photometer (Model 410, Sherwood, England) [35].

For antioxidant enzyme extractions, 0.5 g of fresh roots was homogenized with 50 mM potassium phosphate buffer (pH 7.8), containing 1 mM EDTA, 3 mM 2-mercaptoethanol, and 2% (w/v) polyvinyl-poly-pyrrolidone. The filtered homogenate was then centrifuged at 15,000 g for 30 min at 4 °C, and the resulting supernatant was used to evaluate the activity of superoxide dismutase (SOD), catalase (CAT), and peroxidase (POD). All enzyme activities were measured at 25 °C by an UV-B spectrophotometer (UV-B 2501, Shimadzu, Japan). SOD activity was assayed by monitoring the inhibition of photochemical reduction of nitro blue tetrazolium (NBT) using the method of Beauchamp and Fridovich [37]. One unit of SOD activity was defined as the amount of enzyme required to cause 50% inhibition of NBT reduction. POD activity was determined as described by Hemeda and Kelin [38] using guaiacol as a substrate. One unit of POD activity was defined as the amount of enzyme that increased the absorbance at 470 nm by 0.001 absorbance unit per min. CAT activity was estimated by monitoring the disappearance of H_2_O_2_ at 240 nm [39]. Membrane lipid peroxidation was recorded by the spectrophotometric determination of malondialdehyde using thiobarbituric acid [36]. The soluble protein content was measured as described by Bradford [40] and bovine serum albumin was used as a standard. Total soluble sugar was estimated from the glucose standard curve according to Dubois et al. [41] Nitrate reductase was determined according to Su [42].

Rapeseed were harvested from each pot at maturity in May 2021 to determine the grain yield and other yield-related agronomic traits, including plant height (H), effective branching height (EBH), main sequence height (MSH), one effective branches (OEB), one effective pods (OEP), main sequence pods (MSP), grains per pod (GP) and thousand-grain weight (TGW) were determined simultaneously.

### Statistical analysis

One-way analysis of variance (ANOVA), with different tillage measures as the one fixed factor, was used to assess variations in each indicator. Differences between all treatments were detected using least significant difference (LSD) testing at the 0.05 significance level. Pearson’s correlations were used to analyzed the correlations among indicators. All statistical analyses were conducted using SPSS version 17.0 (SPSS Inc., Chicago, IL, USA).

## Data Availability

The datasets generated during the current study are available from the first author on reasonable request.

## References

[1] Nima D, Mahmoud T, Mostafa V, Ali B (2018). The proteome response of salt-sensitive rapeseed (*Brassica napus* L.) genotype to salt stress. Not Bot Horti Agrobo.

[2] Zhang J, Yang Z, Chen S (2022). Analysis of rapeseed production costs and benefits in Sichuan Province. Sichuan Agr Sci Tech.

[3] Wang Z, Han Y, Luo S, Rong X, Song H, Jiang N, Li C, Yang L (2022). Calcium peroxide alleviates the waterlogging stress of rapeseed by improving root growth status in a rice-rape rotation field. Front Plant Sci.

[4] Cheng P, Feng L, Zhang S, Li L, Guan R, Long W, Xian Z, Zhang J, Shen W (2022). Ammonia borane positively regulates cold tolerance in *Brassica napus* via hydrogen sulfide signaling. BMC Plant Biol.

[5] Li J, Xie T, Chen Y, Zhang Y, Wang C, Jiang Z, Yang W, Zhou G, Guo L, Zhang J. High-throughput UAV-based phenotyping provides insights into the dynamic process and genetic basis of rapeseed waterlogging response in the field. J Exp Bot. 2022;73(15):5264-78. 10.1093/jxb/erac242 35641129

[6] Ding L, Liu R, Li T, Li M, Liu X, Wang W, Yu Y, Cao J, Tan X (2022). Physiological and comparative transcriptome analyses reveal the mechanisms underlying waterlogging tolerance in a rapeseed anthocyanin-more mutant. Biotechnol Biofuels Bioprod.

[7] Men S, Chen H, Chen S, Zheng S, Shen X, Wang C, Yang Z, Liu D (2020). Effects of supplemental nitrogen application on physiological characteristics, dry matter and nitrogen accumulation of winter rapeseed (*Brassica napus* L.) under waterlogging stress. Sci Rep.

[8] Cisse E, Huang J, Li D, Miao L, Xiang L, Yang F (2023). Exogenous spermidine alleviated waterlogging damages in two varieties of *camellia oleifera*. Forests..

[9] Gong J, Zheng Z, Liu Y, Peng S, Jing Y, Chen T, Zhou Q, Li J (2022). Effects of deep ploughing with powder ridge and ditching drainage on soil nutrients and growth and development of flue-cured tobacco. J Northwest A&F Univer.

[10] Ren B, Dong S, Liu P, Zhao B, Zhang J (2016). Ridge tillage improves plant growth and grain yield of waterlogged summer maize. Agr Water Manage.

[11] Du X, He W, Wang Z, Xi M, Xu Y, Wu W, Gao S, Liu D, Lei W, Kong L (2021). Raised bed planting reduces waterlogging and increases yield in wheat following rice. Field Crop Res.

[12] Suo M, Wu J, Liu F, Xu T, Su T, Zhang G, Xiong J (2022). Effects of different water and fertilizer management and tillage practices on soil nutrients and rice yield in post-flooded rice fields.

[13] Wakchaure G, Minhas P, Kumar S, Khapte P, Rane J, Reddy K. Bulb productivity and quality of monsoon onion (Allium cepa L.) as affected by transient waterlogging at different growth stages and its alleviation with plant growth regulators. Agr Water Manage. 2023;278:108136.

[14] Ma Q, Zuo X, Hu C, Cheng L, Li T (2021). Effects of waterlogging on photosynthetic characteristics and yield of summer peanut. J Appl Meteorol.

[15] Ren B, Hu J, Liu P, Zhao B, Zhang J (2021). Responses of nitrogen efficiency and antioxidant system of summer maize to waterlogging stress under different tillage. PeerJ..

[16] Li Z (2019). The difference of soil temperature and humidity, and yield under different cultivation modes of rapeseed.

[17] Tian X, Li Z, Liu Z, Wang Y, Li B, Zhang K, Xu Q, Wang L (2022). Combined effect of biochar and nitrogen fertilizer reduction on rapeseed productivity and nitrogen use efficiency. Arch Agron Soil Sci.

[18] Li D, Miao L, Cisse E, Li L, Chen B, Yang F. Dissecting the below and aboveground specific responses of two waterlogging tolerant arbor species to nutrient supply under waterlogging conditions. Tree Physiol. 2022;43(3):390-403. 10.1093/treephys/tpac127 36300499

[19] Motarjemi S, Styczen M, Petersen R, Jensen K, Plauborg F (2023). Effects of different drainage conditions on nitrogen losses of an agricultural sandy loam soil. J Environ Manag.

[20] Wang Q, Li F, Zhao X, Zhao W, Zhang D, Zhou X, Sample D, Wang X, Liu Q, Li X, Li G, Wang H, Zhang K, Chen J (2022). Runoff and nutrient losses in alfalfa (*Medicago sativa* L) production with tied-ridge-furrow rainwater harvesting on sloping land. Int Soil Water Conse.

[21] Gurpreet K, Gurbir S, Peter P, Kelly A, John M, Bobby R (2020). Management practices to reduce crop production and nitrogen losses from waterlogging. Crop Soils.

[22] Gurpreet K, Brendan A, Kelly A, Peter P, Christopher J (2017). Soil waterlogging and nitrogen fertilizer management effects on corn and soybean yields. Agric J.

[23] Li Q, Zhou S, Liu W, Zhai Z, Pan Y, Liu C, Chern M, Wang H, Huang M, Zhang Z, Tang J, Du H (2021). A chlorophyll a oxygenase 1 gene *ZmCAO1* contributes to grain yield and waterlogging tolerance in maize. J Exp Bot.

[24] LeónBurgos A, Unigarro C, BalagueraLópez H (2022). Soil waterlogging conditions affect growth, water status, and chlorophyll “a” fluorescence in coffee plants (*Coffea arabica* L.). Agronomy..

[25] Sharma S, Bhatt U, Sharma J, Darkalt A, Mojski J, Soni V. Effect of different waterlogging periods on biochemistry, growth, and chlorophyll a fluorescence of *Arachis hypogaea* L. Front Plant Sci. 2022;13:1006258. 10.3389/fpls.2022.1006258 PMC9686000 36438100

[26] Bansal R, Sharma S, Tripathi K, Gayacharan KA (2019). Waterlogging tolerance in black gram [*Vigna mungo* (L.) Hepper] is associated with chlorophyll content and membrane integrity. Indian J Biochem Biophys.

[27] Ren B (2018). Physiological mechanism and regulation of flooding affecting the growth and development of summer maize.

[28] Hasanuzzaman M, Bhuyan M, Zulfiqar F, Raza A, Mohsin S, Mahmud J, Fujita M, Fotopou V (2020). Reactive oxygen species and antioxidant defense in plants under abiotic stress: revisiting the crucial role of a universal defense regulator. Antioxidants..

[29] Pan J, Sharif R, Xu X, Chen X (2021). Mechanisms of waterlogging tolerance in plants: research progress and prospects. Front Plant Sci.

[30] Alscher P, Erturk N, Heath L (2002). Role of superoxide dismutases (SODs) in controlling oxidative stress in plants. J Exp Bot.

[31] Mittler R (2002). Oxidative stress, antioxidants and stress tolerance. Trends Plant Sci.

[32] Gao M, Chen Y, Zhao Y, Wang Y. Sex-specific physiological and biochemical responses of Litsea cubeba under waterlogging stress. Environ Exp Bot. 2022;202:105018.

[33] Ali R, Mohammad-Eghbal G, Saeid J, Mokhtar G, Mohsen S. Impacts of waterlogging on shoot apex development and recovery effects of nitrogen on grain yield of wheat. Euro J Exp Bio. 2012;2(4):1000-7.

[34] Tuo Y, Wang Z, Ya Z, Shi X, Liu X, Ding M, Yang Q (2023). Effect of water and fertilizer regulation on the soil microbial biomass carbon and nitrogen, enzyme activity, and saponin content of *Panax notoginseng*. Agr water. Manage..

[35] Fu J, Luo Y, Sun P, Gao J, Zhao D, Yang P, Hu T (2020). Effects of shade stress on turf grasses morphophysiology and rhizosphere soil bacterial communities. BMC Plant Biol.

[36] Cai Z, Gao Q (2020). Comparative physiological and biochemical mechanisms of salt tolerance in five contrasting highland quinoa cultivars. BMC Plant Biol.

[37] Beauchamp C, Fridovich I (1971). Superoxide dismutase: improved assays and an assay applicable to acrylamide gels. Anal Biochem.

[38] Hemeda H, Kelin B (1990). Effects of naturally occurring antioxidants on peroxidase activity of vegetable extracts. J Food Sci.

[39] Aebi H (1984). Catalase in vitro. Methods Enzymol.

[40] Bradford M (1976). A rapid and sensitive method for the quantitation of microgram quantities of protein utilizing the principle of protein–dye binding. Anal Biochem.

[41] Dubois M, Gilles K, Hamilton J, Rebers P, Smith F (1956). Colorimetric method for determination of sugar and related substances. Anal Chem.

[42] Su P, Gao C, Zhang X, Zhang D, Liu X, Xiang T, Luo Y, Chu K, Zhang G, Bu N, Li Z (2023). Microplastics stimulated nitrous oxide emissions primarily through denitrification: a meta-analysis. J Hazard Mater.

